# Long-range white matter fibres and post-stroke verbal and non-verbal cognition

**DOI:** 10.1093/braincomms/fcae262

**Published:** 2024-08-16

**Authors:** Rebecca W Roth, Deena Schwen Blackett, Ezequiel Gleichgerrcht, Janina Wilmskoetter, Chris Rorden, Roger Newman-Norlund, Souvik Sen, Julius Fridriksson, Natalie Busby, Leonardo Bonilha

**Affiliations:** Department of Neurology, Emory University, Atlanta, GA 30329, USA; Department of Otolaryngology, College of Medicine, Medical University of South Carolina, Charleston, SC 29425, USA; Department of Neurology, Emory University, Atlanta, GA 30329, USA; Department of Health and Rehabilitation Sciences, College of Health Professions, Medical University of South Carolina, Charleston, SC 29425, USA; Department of Psychology, University of South Carolina, Columbia, SC 29208, USA; Department of Psychology, University of South Carolina, Columbia, SC 29208, USA; Department of Neurology, University of South Carolina, Columbia, SC 29209, USA; Department of Communication Sciences and Disorders, University of South Carolina, Columbia, SC 29208, USA; Department of Communication Sciences and Disorders, University of South Carolina, Columbia, SC 29208, USA; Department of Neurology, University of South Carolina, Columbia, SC 29209, USA

**Keywords:** aphasia, white matter, stroke, cognition

## Abstract

Among stroke survivors, linguistic and non-linguistic impairments exhibit substantial inter-individual variability. Stroke lesion volume and location do not sufficiently explain outcomes, and the neural mechanisms underlying the severity of aphasia or non-verbal cognitive deficits remain inadequately understood. Converging evidence supports the idea that white matter is particularly susceptible to ischaemic injury, and long-range fibres are commonly associated with verbal and non-verbal function. Here, we investigated the relationship among post-stroke aphasia severity, cognition, and white matter integrity. Eighty-seven individuals in the chronic stage of stroke underwent diffusion MRI and behavioural testing, including language and cognitive measures. We used whole-brain structural connectomes from each participant to calculate the ratio of long-range fibres to short-range fibres. We found that a higher proportion of long-range fibres was associated with lower aphasia severity, more accurate picture naming, and increased performance on non-verbal semantic memory/processing and non-verbal reasoning while controlling for lesion volume, key damage areas, age, and years post stroke. Our findings corroborate the hypothesis that, after accounting for age and lesion anatomy, inter-individual differences in post-stroke aphasia severity, verbal, and non-verbal cognitive outcomes are related to the preservation of long-range white matter fibres beyond the lesion.

## Teaser

This study evaluated the relationship between post-stroke aphasia severity, cognition, and white matter integrity in 87 chronic stroke survivors. Higher proportions of long-range white matter fibres were found to correlate with reduced aphasia severity and improved verbal and non-verbal cognitive performance, underscoring the importance of long-range fibre preservation in post-stroke language and cognitive outcomes.

## Graphical Abstract

**Graphical abstract:**
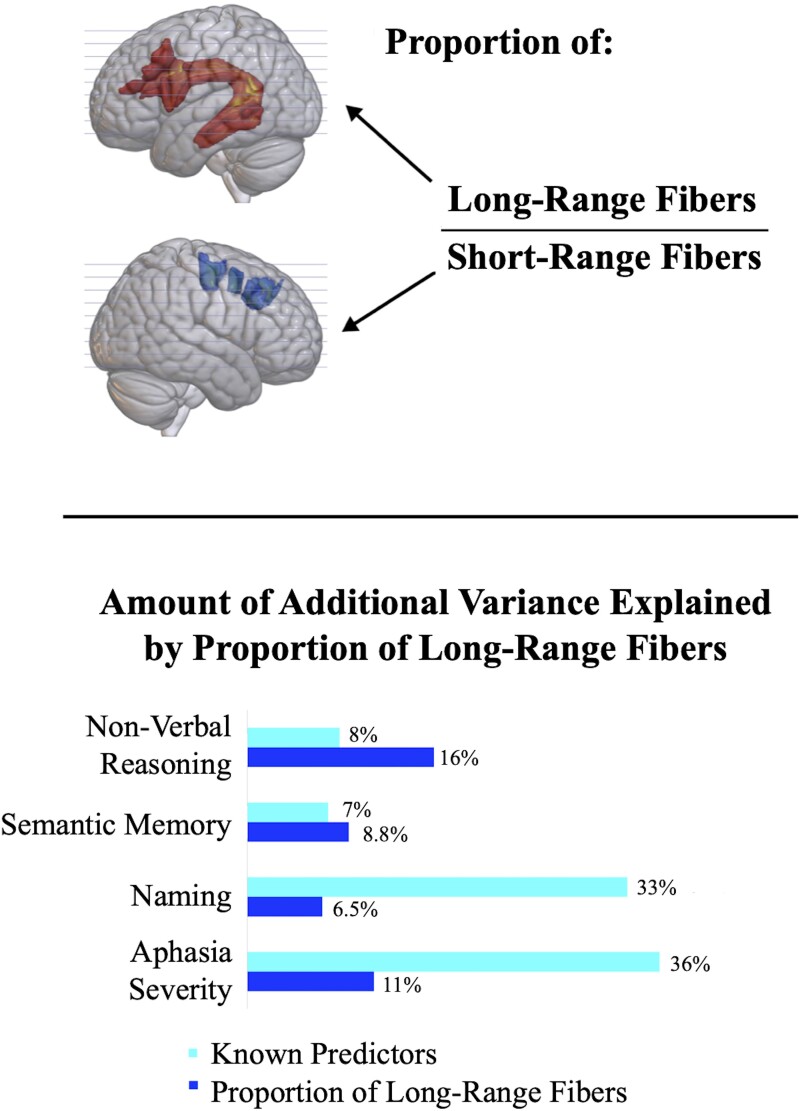

## Introduction

Following stroke, approximately one-third of individuals develop aphasia,^1^ and although many recover in the acute stage, some persist with chronic (>6 months) language and cognitive deficits. Recent evidence suggests that lesion characteristics and age can account for up to 50% of the variance in chronic stroke deficits^2^ and are strong predictors of language recovery following treatment.^3^ However, there remains a large proportion of unexplained variance in aphasia severity, suggesting that the severity of chronic deficits may depend on additional variables.

The degree of preservation of brain networks beyond the lesion is likely a key determinant of aphasia severity or recovery.^4,5^ For example, Basilakos and colleagues demonstrated that damage to the uncinate fasciculus predicted deficits in language fluency.^6^ Similarly, a study employing connectome-based lesion symptom mapping (LSM), a method used to map structural connections across the whole brain, demonstrated that preservation of frontotemporal connections was associated with less severe aphasia.^4^ In addition, preservation of long-range white matter connections was associated with better cognition in general.^7^ This is a crucial mechanistic observation since white matter is more susceptible to ischaemic injury^8^ compared with gray matter. Moreover, among white matter fibres, long-range fibres are more prone to injury due to their elevated metabolic requirements necessary for maintaining structural integrity in contrast to shorter fibres.^9,10^ As such, white matter integrity is particularly at risk in the context of cardiovascular risk factors such as hypertension^11^ and diabetes,^12^ which are prevalent among stroke survivors.

Long-range fibres are known to be related to aphasia severity (as measured by the Western Aphasia Battery-Revised)^13^ and naming recovery [as measured by the Philadelphia Naming Test (PNT)].^14^ However, it remains largely unclear if white matter integrity, specifically preservation of long-range fibres, is associated with non-verbal cognition among stroke survivors with aphasia. Our study seeks to (i) replicate the finding by Wilmskoetter *et al*.^13^ demonstrating that fibre length predicts aphasia severity and (ii) examine measures of semantic memory/processing and non-verbal cognition with similar methods while accounting for factors already shown to be predictive of performance (i.e., age, years post stroke, lesion volume, and damage to key areas). Our objective was to assess the extent of unexplained variability in linguistic and non-verbal factors that could be further elucidated by the integrity of long-range fibres, given that long-range fibres are particularly at risk among stroke survivors. We hypothesized that a higher proportion of long-range fibres would be associated with less severe aphasia and better performance on naming, non-verbal semantic memory/processing, and non-verbal reasoning tasks.

## Materials and methods

Data were collected at the University of South Carolina and at the Medical University of South Carolina. ASHA-certified speech-language pathologists with experience working with individuals with aphasia administered all assessments. The study was approved by the Institutional Review Boards at each university, and informed consent was obtained following guidelines outlined in the Declaration of Helsinki. All participants (*N* = 87) had chronic post-stroke aphasia (>12 months post stroke) and were part of the **P**redicting **O**utcomes of **La**nguage **R**ehabilitation in aphasia (POLAR) clinical trial^15^ (see Kristinsson *et al*., 2021 for more details). Eligible participants did not have a history of other neurological disorders, had a left-hemisphere ischaemic or haemorrhagic stroke, were between 21 and 80 years of age, and were able to provide written or verbal consent. We included participants who had diffusion imaging in the current study. The participants used in Wilmskoetter *et al*. 2019^13^ and Roth *et al*. 2023^14^ are taken from the same large study (POLAR) but in the current paper, we focus on verbal and non-verbal cognitive tasks, expanding previous work to examine the role of semantic memory and non-verbal reasoning.

### Behavioural assessments

Participants completed extensive baseline language testing, including the WAB-R to identify aphasia subtype.^16^ The PNT,^17^ the Pyramids and Palm Trees Test (PPTT),^18^ and matrix reasoning subtest from the Weschler Adult Intelligence Scale – Fourth Edition^19^ were also administered to assess picture naming, semantic memory/processing, and non-verbal reasoning, respectively.

### MRI acquisition and analyses

High-resolution structural MRI data were obtained from all participants using a Siemens Trio 3T (12-channel head coil) or Siemens Prisma 3T (20-channel head/neck coil) scanner located at the Medical University of South Carolina or at the University of South Carolina, respectively. The parameters were as follows:

**T_1_-weighted**: MR-RAGE sequence with 1 mm3 isotropic voxels, FOV matrix of 256 × 256 mm, 9° flip angle, and 192 sagittal slice sequence with TR = 2250 ms, T1 = 925 ms, and TE = 4.15 ms, with parallel imaging (GRAPPA = 2, 80 reference lines)
**T_2_-weighted:** 3D SPACE voxel size of 1 mm^3^, 256 × 256 mm FOV matrix, 160 sagittal slice sequence, variable flip angle, TR = 3,200 ms, TE = 352 ms, with no slice acceleration.
**Diffusion-weighted:** Two echo planar imaging (EPI) scans with 1.5 mm isotropic voxels were acquired for each participant. The sequences were identical except that one was acquired in the Anterior–Posterior direction, and the other was acquired in the Posterior–Anterior phase encoding direction. Both sequences were 42 directions with *b* = 1000 s/mm^2^ (60 volumes) *b* = 2000s/mm^2^ (60) and *b* = 0 s/mm^2^ (11), TR = 5250 ms, TE = 80 ms, 210 × 210 FOV, with parallel imaging GRAPPA = 2, and 80 1.5 mm axial slices, TA = 4.02 s per scan.

**Figure 1:**
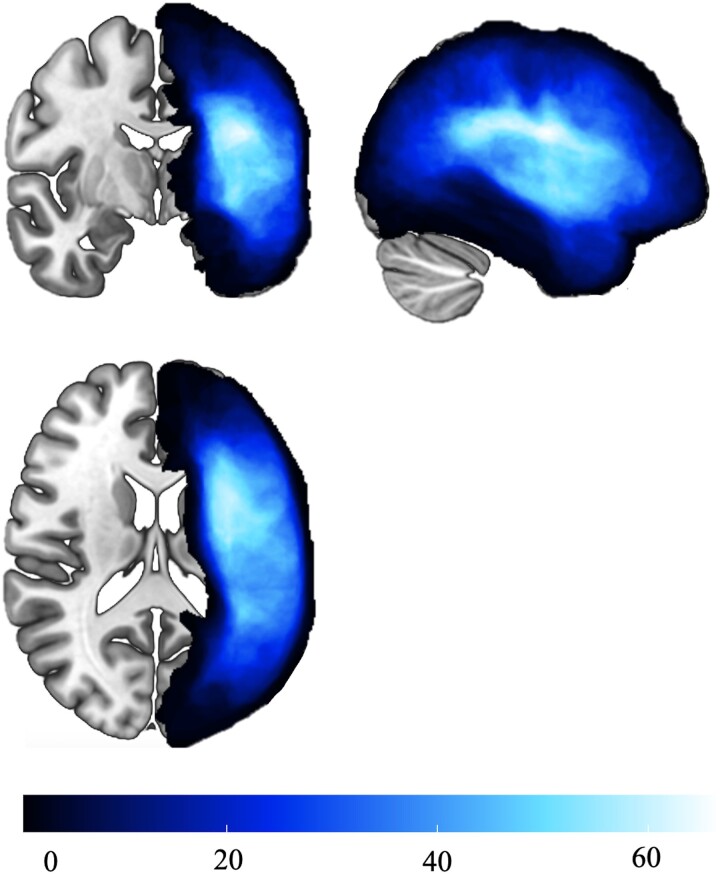
**Lesion overview.** Lesion overlay for 87 individuals with chronic, left-hemisphere stroke, and aphasia used in this study.

Stroke lesions were manually drawn on T2-weighed images by a neurologist or by a trained research staff blinded to the behavioural scores. A stroke-specific image pre-processing pipeline (*nii_*preprocess: https://github.com/neurolabusc/nii_preprocess) was used to normalize stroke lesions into MNI space (see Fig. 1). Briefly, this approach involved enantiomorphic healing of the T_1_-weighed image (via replacement of stroke tissue with healthy tissue from the opposite hemisphere), calculation of parameters for normalizing the healed T_1_ to MNI space, co-registration of the T_2_-weighted and lesion mask to the T_1_-weighted, and application of the application of the spatial transform to the lesion mask.^20^ All processing was done with custom MATLAB scripts (R2017b, The Mathworks; https://zenodo.org/records/4027711) that relied on SPM12 (Functional Imaging Laboratory, Wellcome Trust Centre for Neuroimaging, Institute of Neurology www.fil.ion.ucl.ac.uk/spm]) and FSL software^20^ (v6.0.3). TOPUP and Eddy motion correction were applied to diffusion images with FSL’s toolbox.^21,22^

### Proportion of long-range fibres

We used a whole-brain structural connectome approach to measure white matter network integrity through diffusion tensor imaging employing the following steps: a reverse normalization was performed to warp the Johns Hopkins University (JHU) atlas into standard space using SPM12. Probabilistic grey and white matter maps from T_1_-weighted images were used to guide tractography. We then used FSL's FDT's method (Bedpostx and FDT's probtrackX, 5000 individual pathways drawn through the probability distributions on principal fibre direction, curvature threshold: 0.2, maximum steps: 200, step length: 0.5 mm, and distance correction) to measure pairwise connectivity between all possible grey matter regions in the JHU atlas. The probabilistic white matter map excluding the stroke lesion was used as a waypoint mask. We then computed the number of probable streamlines arriving in one region when another was seeded for each possible pair of regions (averaged with the opposite direction given the undirected property of diffusion tensor imaging). Length and space biases were accounted for by correcting weighted connectivity between regions based on the distance travelled by the streamlines and by the volume of each region. More specifically, tractography was performed using the distance correction function in FSL's Probtrackx and the resulting number of streamlines between regions was divided by the sum of the volumes of the connected regions. These steps yielded an individual connectivity adjacency matrix for each individual. Connections lower than the 20th percentile were considered possibly spurious and thus transformed into zero-weight values.

**Figure 2:**
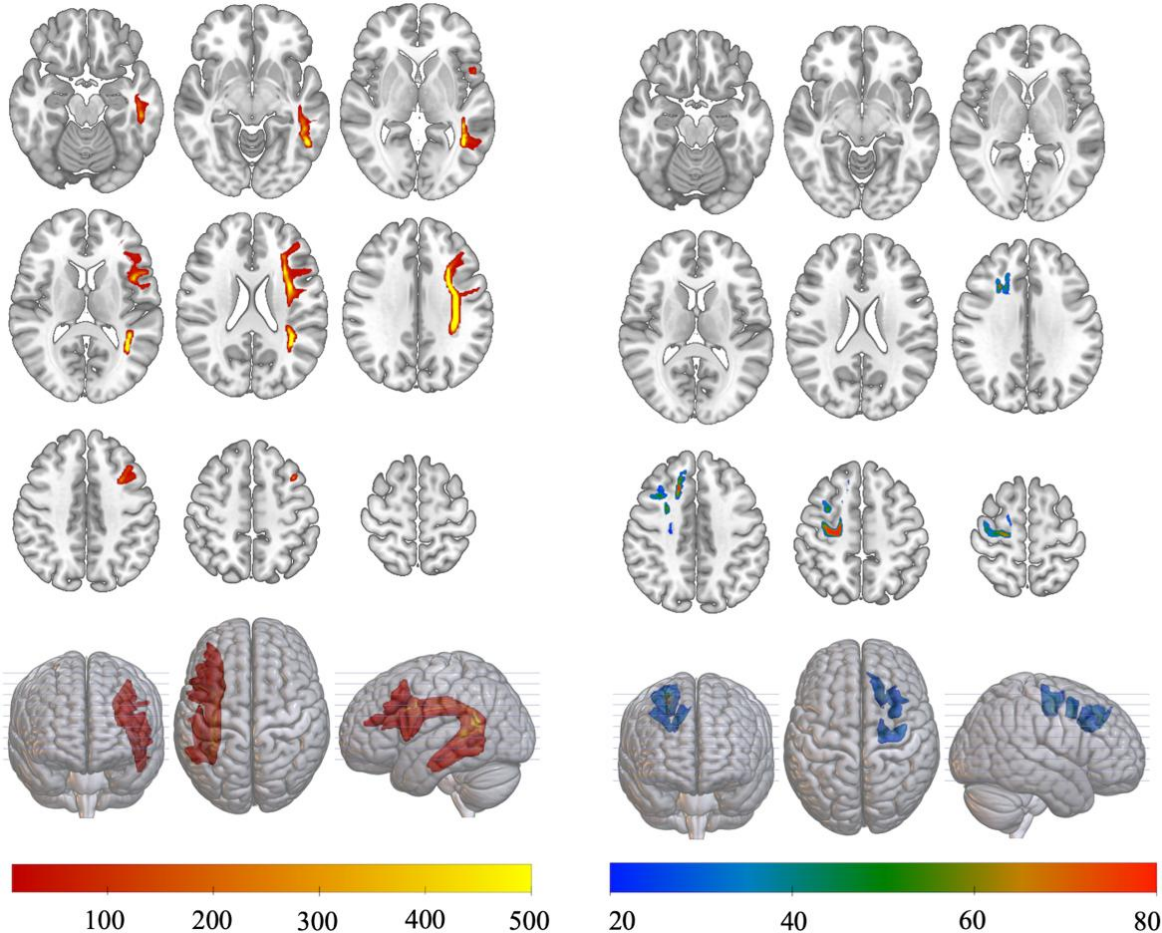
**Short-range versus long-range fibres.** This figure provides a visual representation of example pathways traversed by long-range versus short-range fibres. These are tract density images in standard MNI152 space created in DSI Studio using the Human Connectome Project template from 1065 participants. The data evaluated in this study used probabilistic tractography to reconstruct whole-brain connectomes since probabilistic tractography is more accurate to resolve fibre crossings or complex anatomy. Nonetheless, probabilistic tractography does not allow for the visualization of white matter pathways in a direct manner, which can be demonstrated by deterministic examples of white matter fibre anatomy, as shown here. Thus, this figure is intended to provide a visual aid for the interpretation of the results only. The mosaic on the left demonstrates a long-range association pathway (arcuate fasciculus in the left hemisphere) including only fibres with distance travelled greater than 120 mm. Conversely, the mosaic on the right demonstrates examples of u-fibres in the right hemisphere with distance travelled lower than 20 mm. The examples used in this figure are for the visual representation of long versus short-range fibres.

To quantify the proportion of long-range to short-range fibres, we used a similar approach as in Wilmskoetter *et al*. (2019)^13^ and Roth *et al*. (2023).^14^ We first measured the Euclidean distance between centroids of JHU regions of interest (ROIs) in the Montreal Neurology Institute Space (MNI). Connections whose distance between centroids was within the first quartile (lowest 25%), second or third quartiles, and fourth quartile were categorized as short, medium, and long-range fibres, respectively. We computed the ratio of long-range to short-range fibres (i.e., Number of Long-Range fibres/Number of Short-Range fibres), which we will refer to as the proportion of long-range fibres from this point on (Fig. 2).

### Lesion symptom mapping

To evaluate which regions were associated with language and cognition, we performed a region of interest level univariate LSM^23^ analysis using the NiiStat toolbox (www.nitrc.org/projects/niistat) in MATLAB 2022a. We used the default settings with lesion as the modality, FDR correction with a corrected *P*-value of 0.05, and the JHU atlas. We performed a one-tailed test (assuming that lesions resulted in worse performance). We ran separate models for WAB-R Aphasia Quotient (WAB-AQ), PNT, PPTT, and matrix reasoning. This analysis identified the regions associated with each behavioural variable. We then averaged damage across regions revealed to be significant in LSM per behavioural task and included this as an independent variable in the subsequent analyses. This is denoted as ‘key damaged areas’ from this point on.

### Statistical analyses

All statistical analyses were completed in SPSS. We first ran an analysis of variance (ANOVA) to examine if stroke type was associated with the proportion of long-range fibres to ensure that the stroke type was not a confound that could affect our main analyses. Next, we regressed out the variance caused by lesion volume, age, years post stroke, and damage to key regions in WAB-AQ, PNT, PPTT, and matrix reasoning. We then used Pearson's correlations to examine the relationship between standardized residuals produced by the regression above with the proportion of long-range fibres, correcting for multiple comparisons with a false discovery rate (FDR). This also allowed us to examine the amount of variance predicted by proportion of long-range fibres beyond lesion volume, age, years post stroke, and damage to key brain regions.

## Results

### Participants

Based on the WAB-R, 45% of participants had Broca's aphasia, followed by anomic aphasia (28%), conduction aphasia (15%), Wernicke's aphasia (6%), global aphasia (5%), and trans-cortical motor aphasia (1%).^16^ Participants were on average 60.52 years old (*SD =* 11.24) and 3.87 years post stroke (*SD* = 3.98). Participants were 60% male and 40% female, and the majority experienced an ischaemic stroke (66%), followed by haemorrhagic (26%), and other aetiology (8%), see Table 1 (note ‘other’ aetiology refers to cases in which it was unclear whether the stroke was ischaemic or haemorrhagic).

**Table 1:** Participant variables

Categorical variables	Count
Stroke aetiology (ischaemic, haemorrhagic, other)	57:23:7
Sex (male:female)	52:35
Race (Caucasian:African American)	65:22
Ethnicity (Hispanic:Non-Hispanic)	0:87

Continuous variables	Mean (SD)
Age at testing (years)	60.52 (11.24)
Years post stroke	3.87 (3.98)
Education (years)	15.39 (2.32)
WAB-AQ	59.77 (22.89)
PNT	80.31 (61.80)
PPTT	45.37 (5.12)
Matrix reasoning	11.98 (5.73)
Lesion volume	122,000 (110,622)
Proportion of long-range fibres	0.15 (0.038)

### Stroke aetiology

A one-way ANOVA was conducted to compare the proportion of long-range fibres and stroke aetiology to ensure stroke type was not a confound that could affect our main analyses. Proportion of long-range fibres across did not significantly differ as a function of stroke type, *F*(84, 2) = 0.89, *P* = 0.41. Thus, we stroke aetiology was not considered in subsequent analyses.

### LSM results

Nine left-hemisphere regions were significantly associated with the WAB-AQ: post-central gyrus, pre-central gyrus, inferior temporal gyrus, superior corona radiata, posterior corona radiata, retrolenticular part of the internal capsule, external capsule, superior longitudinal fasciculus, and posterior insula. For the PNT, three regions were significant in the left hemisphere: superior corona radiata, posterior corona radiata, and superior longitudinal fasciculus (see Table 2 for mean damage). No regions were significant for PPTT or matrix reasoning.

**Table 2:** Average damage to ROI significant in LSM for WAB-AQ and PNT

Region of interest	Mean (*SD*)
Post-central gyrus	0.25 (0.27)
Pre-central gyrus	0.29 (0.27)
Inferior temporal gyrus	0.10 (0.21)
Superior corona radiata^a^	0.39 (0.33)
Posterior corona radiata^a^	0.27 (0.29)
Retrolenticular internal capsule	0.27 (0.28)
External capsule	0.50 (0.40)
Superior longitudinal fasciculus^a^	0.52 (0.30)
Posterior insula	0.55 (0.39)

^a^Regions significant for PNT as all regions were significant for WAB-AQ.

### Regression of covariates

To control for predictors besides white matter connectivity before examining the relationship between behaviour and proportion of long-range fibres, we regressed the behavioural dependent variable from covariates that have been shown to predict aphasia severity and cognition: age, years post stroke, lesion volume, and damage to key regions. These covariates explained approximately 36% of the variance in WAB-AQ (*R =* 0.62, adjusted *R*^2^ = 0.358), 33% of the PNT (*R =* 0.60, adjusted *R*^2^ = 0.33), 7% of the PPTT (*R =* 0.32, adjusted *R*^2^*=* 0.071), and 8% of matrix reasoning (*R =* 0.34, adjusted *R*^2^*=* 0.081). The full regression results can be seen in Table 3. Next, we correlated the residuals saved from this regression with the proportion of long-range fibres to obtain the amount of variance the proportion of long-range fibres could predict beyond established covariates.

**Table 3:** Regression of covariates

	WAB-AQ	PNT	PPTT	Matrix reasoning
Model statistics	*F* = 12.99, *P =* 0.002	*F* = 11.72, *P =* 0.002	*F* = 3.21, *P =* 0.027	*F* = 3.54, *P =* 0.024
Age	*t* = −2.71, *P =* 0.008	*t* = −3.06, *P =* 0.003	*t* = 0.16, *P =* 0.87	*t* = −2.89, *P =* 0.005
Years post stroke	*t* = 1.84, *P* = 0.069	*t* = 2.91, *P =* 0.005	*t* = 1.71, *P =* 0.09	*t* = 1.28, *P =* 0.203
Damage to key ROIs	*t* = −3.14, *P =* 0.002	*t* = −3.45, *P <* 0.001	*N/A*	*N/A*
Lesion volume	*t* = −1.63, *P =* 0.106	*t* = −2.37, *P =* 0.02	*t* = −2.72, *P =* 0.008	*t* = −1.40, *P =* 0.16

### Proportion of long-range fibres and behavioural measures

**Figure 3:**
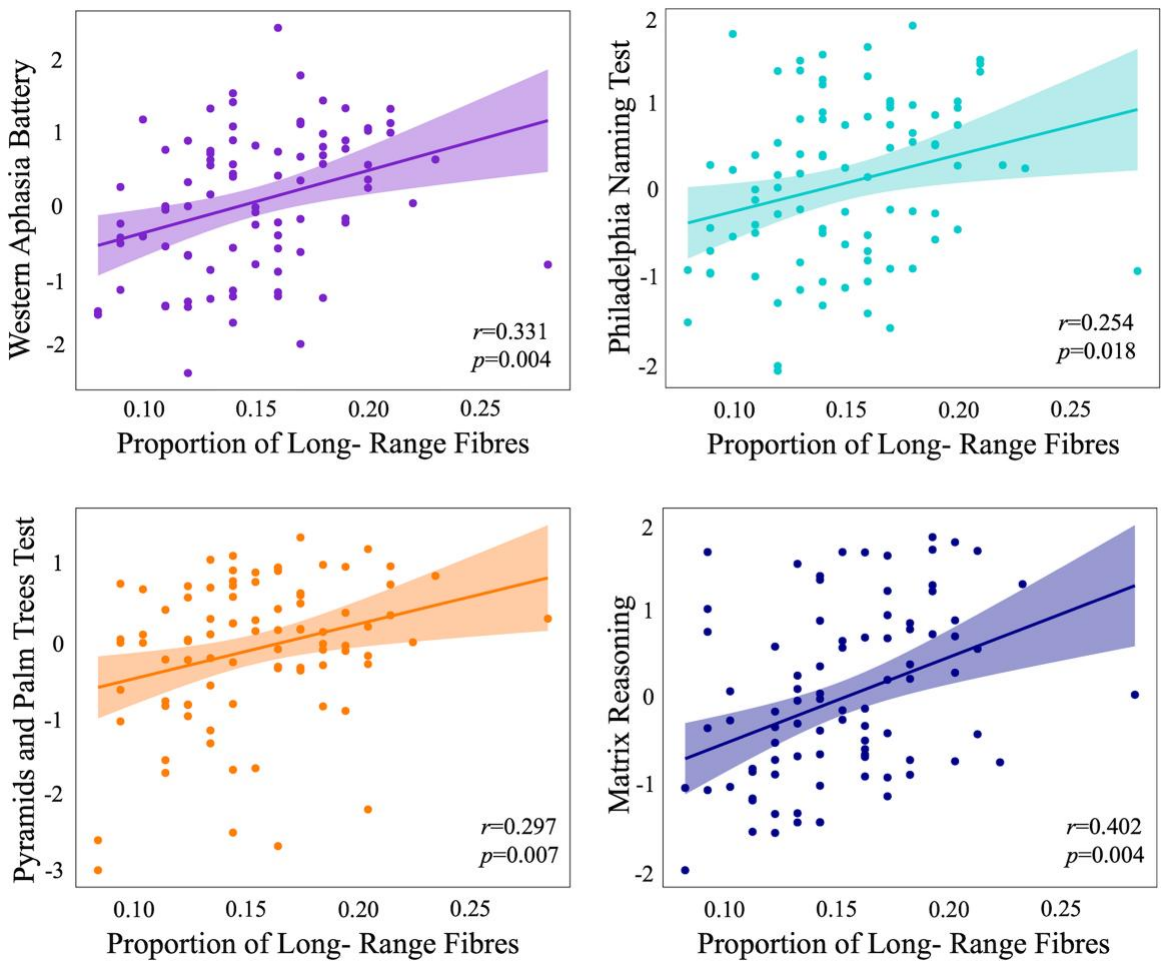
**Scatterplots of proportion of long-range fibres and behavioural measures.** Note all *P*-values are corrected for multiple comparisons.

**Figure 4:**
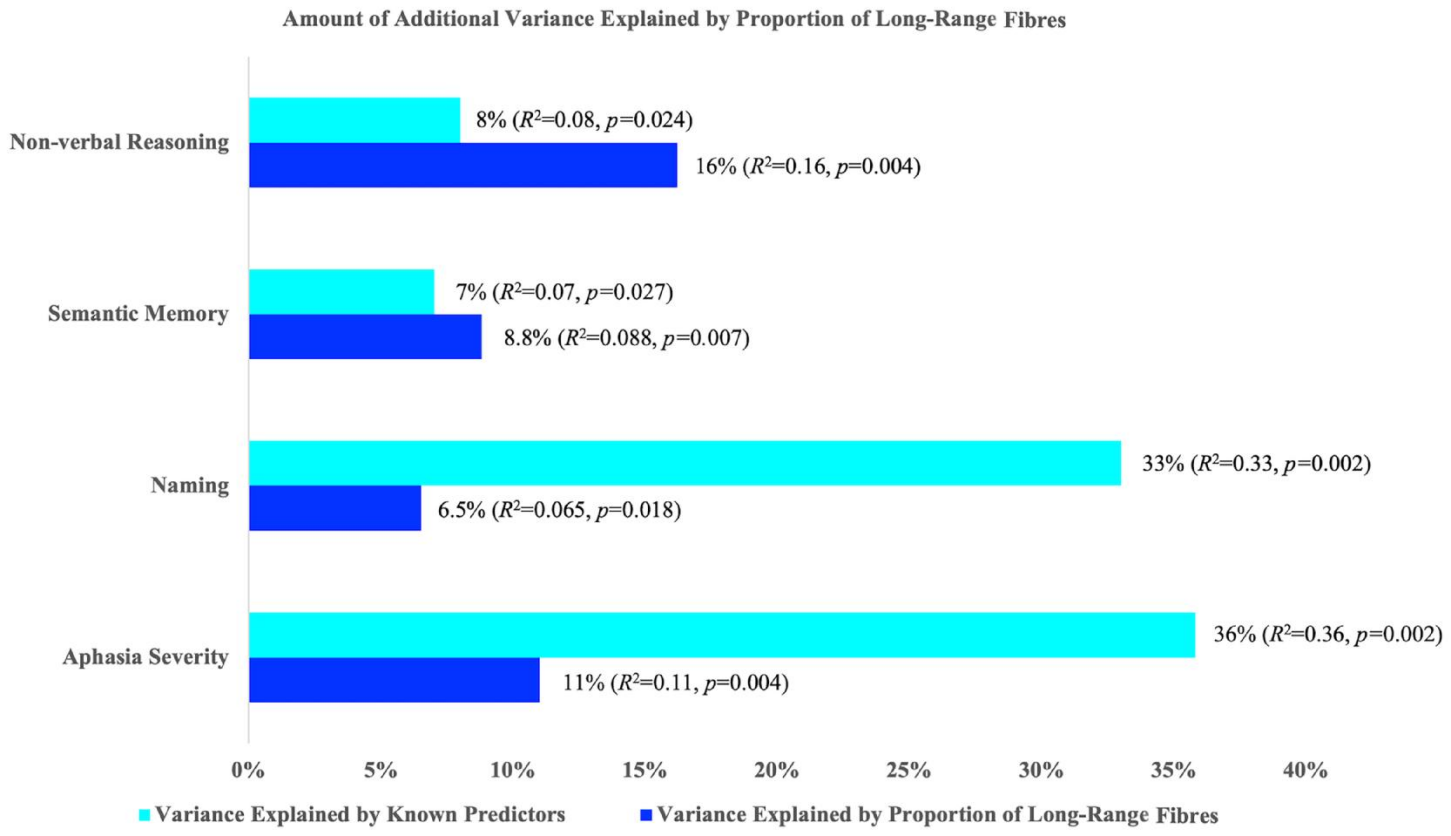
**Additional variance explained in behavioural measures by the proportion of long-range fibres, specifically for language (WAB-AQ, PNT), semantic memory/processing (PPTT), and non-verbal cognitive measures (matrix reasoning).** Note all *P*-values are corrected for multiple comparisons.

The proportion of long-range fibres explained an additional 11.0% of the variance in WAB-AQ (*R* = 0.331, *R*^2^*=* 0.11, corrected *P* = 0.004), 6.5% of variance in the PNT (*R* = 0.254, *R*^2^*=* 0.065, corrected *P* = 0.018), 8.8% of the variance in PPTT (*R* = 0.297, *R*^2^*=* 0.088, corrected *P* = 0.007), and 16.2% of the variance in matrix reasoning (*R* = 0.402, *R*^2^*=* 0.162, corrected *P* = 0.004), see Figs 3 and 4.

## Discussion

This study investigated the relationship among proportion of long-range fibres and aphasia severity, language, and cognition in post-stroke aphasia. We observed that proportion of long-range fibres explained variance in aphasia severity, language, and cognition beyond age, years post stroke, lesion volume, and damage to key regions. Specifically, a higher proportion of long-range fibres predicted decreased aphasia severity, as well as increased performance on naming, semantic memory/processing, and non-verbal reasoning tasks.

### Importance of long-range fibres

These results corroborate previous findings suggesting that the integrity (defined here as the proportion of long-range fibres) of residual white matter tissue beyond the lesion contributes to chronic aphasia severity even when controlling for lesion volume and damage to key ROI.^4,6,24^ Given that white matter tracts are particularly susceptible to injury and the prevalence of cardiovascular risk factors in stroke survivors, structural connections may be particularly important for cognition in this population.

The results here extend this notion by demonstrating that the increased proportion of long-range fibres not only affects aphasia severity, but also semantic memory/processing and non-verbal reasoning. Prior work in other neurological conditions has demonstrated the clinical relevance of long-range fibres, by showing that long-range fibres can be affected in Alzheimer’s disease^25^ and multiple sclerosis.^26^ Long-range fibres have also been shown to support verbal intelligence in healthy aging and to be associated with lower global efficiency,^27^ a graph theory measure that has been used frequently in the literature and is linked to language comprehension.^28^ Similarly, shorter fibre length may be linked to executive dysfunction in healthy aging, and the relative frequency of short-range fibres may increase with age.^29^

Interestingly, our lesion mapping results yielded no significant regions of interests associated with matrix reasoning. These findings are in accordance with neuropsychological models suggesting that frontal lesions can be associated with non-verbal reasoning deficits, since the majority of individuals included in the current study had primarily peri-Sylvian fissure lesions due to middle cerebral artery stroke (Fig. 1). Additionally, a study by Fonseca and colleagues examined a variety of cognitive measures in chronic post-stroke aphasia, and scores on the matrix reasoning subset of the WAIS-IV did not differ between individuals with chronic aphasia and controls who experienced a left-hemisphere stroke but did not develop aphasia,^30^ although both groups performed worse than healthy controls.^31^ Importantly, our results demonstrate that proportion of long-range fibres was a significant predictor of matrix reasoning scores, suggesting that for behavioural deficits which cannot be explained by the lesion, white matter connections may be critical. It should be also noted that the lack of association between lesion volume and the WAB-R in the multiple regression model was likely due to the inter-relationship between lesion volume and damage to key regions, which were entered in the same model. As such, the collinearity between these variables eliminated the effect of global lesion volume, which is often directly associated with aphasia severity.

Additionally, age was not a significant predictor semantic memory/processing. Many studies have shown that semantic processing is relatively preserved with aging; however, a reduction in semantic memory after 70 years of age has also been observed.^32^ Similarly, language comprehension relies on semantic processing/memory.^33^ As our participants ranged between 29 and 80 years of age, this may explain why we did not see an age effect on tasks relying on semantic memory (PPTT). Despite age not being a significant predictor of PPTT scores, proportion of long-range fibres was a significant predictor over what can be explained by the lesion. Future studies could investigate where in the brain long-range fibres are most important (e.g., perilesional hemisphere, contralesional hemisphere, frontal, temporal, etc.). Notably, recent literature on the semantic cognition suggests that semantic control is the process by which information stored in semantic memory is retrieved.^34,35^ Therefore, performance on the PPTT likely captures both semantic knowledge and semantic control abilities. Of note, all participants in this study were in the chronic stage of aphasia recovery, when their symptoms are expected to have plateaued. For this reason, years post stroke was not strongly associated with measures beyond naming.

### Limitations

There are several limitations to the current study. First, we focused on a few measures of cognition thought to be largely independent of the language system, since measures of cognition can be affected by aphasia severity. This is a relatively limited aspect of cognition. In addition, our analyses did not control for years of education. It is possible that years of education may influence the relationship between brain structure and cognition, but this was not directly tested.

Another limitation is the fact that this is whole-brain connectome study, which did not directly focus on specific pathways, but rather on global proportions. As such, it did not directly test which long-range fibres may be more important for the behavioural measures studied. Similarly, fibre length is only one measure of structural network integrity and future work could include other diffusion metrics, such as fractional anisotropy (FA) or mean diffusivity (MD) and their relationships with behaviour.

## Conclusions

Our results highlight the role of the proportion of long-range fibres in post-stroke aphasia as a predictor of severity and cognition. A higher proportion of long-range white matter fibres provided further explanatory value to the variance in aphasia severity, language, and cognition beyond age, years post stroke, lesion volume, and damage to key regions.

## Data Availability

The data and analysis scripts that support the findings of this study are available upon request to the first and corresponding author. The requestor should have IRB approval at the local institution to evaluate these data and a data user agreement prior to sharing the data is necessary. Both nii_preprocess and NiiStat are available here: https://github.com/neurolabusc/nii_preprocess; www.nitrc.org/projects/niistat.

## References

[1] Berthier ML . Poststroke aphasia: Epidemiology, pathophysiology and treatment. Drugs Aging. 2005;22:163–182. 15733022 10.2165/00002512-200522020-00006

[2] Johnson L , Nemati S, Bonilha L, et al Predictors beyond the lesion: Health and demographic factors associated with aphasia severity. Cortex. 2022;154:375–389. 35926368 10.1016/j.cortex.2022.06.013 PMC11205278

[3] Busby N , Hillis AE, Bunker L, et al Comparing the brain–behaviour relationship in acute and chronic stroke aphasia. Brain Commun. 2023;5:fcad014. 37056476 10.1093/braincomms/fcad014 PMC10088484

[4] Fridriksson J , den Ouden DB, Hillis AE, et al Anatomy of aphasia revisited. Brain. 2018;141:848–862. 29360947 10.1093/brain/awx363 PMC5837461

[5] Busby N , Wilmskoetter J, Gleichgerrcht E, et al Advanced brain age and chronic poststroke aphasia severity. Neurology. 2022;100:e1166–e1176. 36526425 10.1212/WNL.0000000000201693 PMC10074460

[6] Basilakos A , Fillmore PT, Rorden C, Guo D, Bonilha L, Fridriksson J. Regional white matter damage predicts speech fluency in chronic post-stroke aphasia. Front Hum Neurosci. 2014;8:845. 25368572 10.3389/fnhum.2014.00845 PMC4201347

[7] Hilal S , Liu S, Wong TY, et al White matter network damage mediates association between cerebrovascular disease and cognition. J Cereb Blood Flow Metab. 2021;41:1858–1872. 33530830 10.1177/0271678X21990980 PMC8327109

[8] Hamner MA , Möller T, Ransom BR. Anaerobic function of CNS white matter declines with age. J Cereb Blood Flow Metab. 2011;31:996–1002. 21179073 10.1038/jcbfm.2010.216 PMC3070977

[9] Buzsáki G . Rhythms of the brain. Oxford University Press; 2006.

[10] Ju H , Hines ML, Yu Y. Cable energy function of cortical axons. Sci Rep. 2016;6:29686. 27439954 10.1038/srep29686 PMC4954988

[11] Kelly DM , Rothwell PM. Blood pressure and the brain: The neurology of hypertension. Pract Neurol. 2020;20:100–108. 31558584 10.1136/practneurol-2019-002269

[12] Ryan CM , van Duinkerken E, Rosano C. Neurocognitive consequences of diabetes. Am Psychol. 2016;71:563–576. 27690485 10.1037/a0040455

[13] Wilmskoetter J , Marebwa B, Basilakos A, et al Long-range fibre damage in small vessel brain disease affects aphasia severity. Brain. 2019;142:3190–3201. 31501862 10.1093/brain/awz251 PMC7962907

[14] Roth R , Busby N, Wilmskoetter J, et al Diabetes, brain health, and treatment gains in post-stroke aphasia. Cereb Cortex. 2023:33:8557–8564. 37139636 10.1093/cercor/bhad140 PMC10321080

[15] Kristinsson S , Basilakos A, Elm J, et al Individualized response to semantic versus phonological aphasia therapies in stroke. Brain Commun. 2021;3:fcab174. 34423302 10.1093/braincomms/fcab174 PMC8376685

[16] Kertesz A , Raven JC. WAB-R: Western aphasia battery-revised. In Western aphasia battery. PsychCorp; 2007. doi: 10.1037%2Ft15168-000.

[17] Roach A , Schwartz MF, Martin N, Grewal RS, Brecher A. The Philadelphia naming test: Scoring and rationale. Clin Aphasiol. 1996;24:121–133.

[18] Howard D , Patterson K. The pyramids and palm trees test. Pearson; 1992.

[19] Erdodi LA , Abeare CA, Lichtenstein JD, et al Wechsler adult intelligence scale-fourth edition (WAIS-IV) processing speed scores as measures of noncredible responding: The third generation of embedded performance validity indicators. Psychol Assess. 2017;29:148–157. 27124099 10.1037/pas0000319

[20] Rorden C , McKinnon E, Hanayik T, Yourganov G, Reddy D. Nii_preprocess. Zenodo. DOI release; 2020.

[21] Andersson JLR , Sotiropoulos SN. An integrated approach to correction for off-resonance effects and subject movement in diffusion MR imaging. Neuroimage. 2016;125:1063–1078. 26481672 10.1016/j.neuroimage.2015.10.019 PMC4692656

[22] Bodammer N , Kaufmann J, Kanowski M, Tempelmann C. Eddy current correction in diffusion-weighted imaging using pairs of images acquired with opposite diffusion gradient polarity. Magn Reson Med. 2004;51:188–193. 14705060 10.1002/mrm.10690

[23] Rorden C , Karnath HO, Bonilha L. Improving lesion-symptom mapping. J Cogn Neurosci. 2007;19:1081–1088. 17583985 10.1162/jocn.2007.19.7.1081

[24] Basilakos A , Stark BC, Johnson L, et al Leukoaraiosis is associated with a decline in language abilities in chronic aphasia. Neurorehabil Neural Repair. 2019;33:718–729. 31315507 10.1177/1545968319862561 PMC6693961

[25] Gao J , Cheung RT, Chan YS, Chu LW, Mak HK, Lee TM. The relevance of short-range fibres to cognitive efficiency and brain activation in aging and dementia. PLoS One. 2014;9:e90307. 10.1371/journal.pone.0090307 PMC3973665 24694731

[26] Meijer KA , Steenwijk MD, Douw L, Schoonheim MM, Geurts JJG. Long-range connections are more severely damaged and relevant for cognition in multiple sclerosis. Brain. 2020;143:150–160. 31730165 10.1093/brain/awz355 PMC6938033

[27] Marebwa BK , Adams RJ, Magwood GS, et al Cardiovascular risk factors and brain health: Impact on long-range cortical connections and cognitive performance. J Am Heart Assoc. 2018;7:e010054. 30520672 10.1161/JAHA.118.010054 PMC6405561

[28] Zhu Z , Fan Y, Feng G, Huang R, Wang S. Large scale brain functional networks support sentence comprehension: Evidence from both explicit and implicit language tasks. PLoS One. 2013;8:e80214. 24244653 10.1371/journal.pone.0080214 PMC3823842

[29] Behrman-Lay AM , Usher C, Conturo TE, et al fibre bundle length and cognition: A length-based tractography MRI study. Brain Imaging Behav. 2015;9:765–775. 25376332 10.1007/s11682-014-9334-8 PMC4424188

[30] Fonseca J , Raposo A, Martins IP. Cognitive functioning in chronic post-stroke aphasia. Applied Neuropsychology: Adult. 2019;26:355–364. 29432034 10.1080/23279095.2018.1429442

[31] Fonseca J , Ferreira JJ, Martins IP. Cognitive performance in aphasia due to stroke: A systematic review. Int J Disabil Hum Dev. 2017;16:127–139.

[32] Verhaegen C , Poncelet M. Changes in naming and semantic abilities with aging from 50 to 90 years. J Int Neuropsychol Soc. 2013;19:119–126. 23237304 10.1017/S1355617712001178

[33] Martin RC . The critical role of semantic working memory in language comprehension and production. Curr Dir Psychol Sci. 2021;30:283–291. 34789966 10.1177/0963721421995178 PMC8594863

[34] Chiou R , Humphreys GF, Jung J, Lambon Ralph MA. Controlled semantic cognition relies upon dynamic and flexible interactions between the executive ‘semantic control’ and hub-and-spoke ‘semantic representation’ systems. Cortex. 2018;103:100–116. 29604611 10.1016/j.cortex.2018.02.018 PMC6006425

[35] Jefferies E , Wang X. Semantic cognition: Semantic memory and semantic control. Oxford Research Encyclopedia of Psychology. 2021. https://oxfordre.com/psychology/display/10.1093/acrefore/9780190236557.001.0001/acrefore-9780190236557-e-760

